# Circulating Apoptotic Signals During Acute and Chronic Exposure to High Altitude in Kyrgyz Population

**DOI:** 10.3389/fphys.2019.00054

**Published:** 2019-02-05

**Authors:** Djuro Kosanovic, Simon Maximilian Platzek, Aleksandar Petrovic, Akylbek Sydykov, Abdirashit Maripov, Argen Mamazhakypov, Meerim Sartmyrzaeva, Kubatbek Muratali Uulu, Meerim Cholponbaeva, Aidana Toktosunova, Nazgul Omurzakova, Melis Duishobaev, Christina Vroom, Oleg Pak, Norbert Weissmann, Hossein Ardeschir Ghofrani, Akpay Sarybaev, Ralph Theo Schermuly

**Affiliations:** ^1^Chair for Pulmonary Pharmacotherapy, Member of the German Center for Lung Research, Universities of Giessen and Marburg Lung Center, Giessen, Germany; ^2^Sechenov First Moscow State Medical University (Sechenov University), Moscow, Russia; ^3^Kyrgyz National Centre for Cardiology and Internal Medicine, named after Academician Mirsaid Mirrakhimov, Bishkek, Kyrgyzstan

**Keywords:** high altitude, circulating apoptotic markers, Fas ligand, hypoxic pulmonary hypertension, pulmonary artery smooth muscle cells

## Abstract

**Background:** Circulating apoptotic signals (CASs) have been described in the pathologies associated with dysregulated apoptosis, such as cancer, heart diseases, and pulmonary hypertension (PH). However, nothing is known about the expression profiles of these markers in the circulation of humans exposed to acute and chronic effects of high altitude (HA).

**Methods:** Gene expression levels of different apoptotic signals (ASs) were analyzed in human pulmonary artery smooth muscle cells (PASMCs) upon hypoxia incubation. In addition, we measured the plasma values of relevant CAS in Kyrgyz volunteers during acute and chronic exposure to HA. Finally, we analyzed the effects of pro-apoptotic mediator Fas ligand (FasL) on apoptosis and proliferation of human PASMCs.

**Results:** Several cellular AS were increased in PASMCs exposed to hypoxia, in comparison to normoxia condition. Among analyzed CAS, there was a prominent reduction of FasL in lowlanders exposed to HA environment. Furthermore, decreased circulatory levels of FasL were found in highlanders with HA-induced PH (HAPH), as compared to the lowland controls. Furthermore, FasL concentration in plasma negatively correlated with tricuspid regurgitant gradient values. Finally, FasL exerted pro-apoptotic and anti-proliferative effects on PASMCs.

**Conclusion:** Our data demonstrated that circulating levels of FasL are reduced during acute and chronic exposure to HA environment. In addition, dysregulated FasL may play a role in the context of HAPH due to its relevant functions on apoptosis and proliferation of PASMCs.

## Introduction

High altitude is a well-known extreme environment characterized by hypoxia, among other abiotic factors, and may exert prominent acute and chronic effects on respiratory and cardiovascular systems (Maggiorini and Leon-Velarde, 2003; West, 2012; Mirrakhimov and Strohl, 2016). An important part of the human population inhabits high altitudes of our planet and a large number of people visit these elevations periodically due to several reasons (Mirrakhimov and Strohl, 2016; Azad et al., 2017). Short-term exposure of non-acclimatized people to high altitude may provoke the appearance of several acute mountain disorders, including acute mountain sickness and high-altitude pulmonary edema (West, 2012). Long-term exposure to this challenging external surrounding may lead to development of high altitude-induced pulmonary hypertension (HAPH), which is a pathological condition currently classified in the group 3 of pulmonary hypertension (PH) (Maggiorini and Leon-Velarde, 2003; Simonneau et al., 2013; Mirrakhimov and Strohl, 2016).

In general, pulmonary vascular remodeling as the main attribute of PH pathology appears due to significant dysregulation of normal processes in the pulmonary vascular cells, such as proliferation and apoptosis (Schermuly et al., 2011; Malczyk et al., 2016; Stenmark et al., 2018; Thenappan et al., 2018). Abnormal regulation of apoptosis and occurrence of “apoptotic-resistant” phenotype in pulmonary vascular cells, such as pulmonary artery smooth muscle cells (PASMCs), are important hallmarks of hypoxia-induced PH pathogenesis (Yang et al., 2001; Chen et al., 2014; Li et al., 2017; You et al., 2018). Due to the existence of these cellular abnormalities, e.g., increased proliferation and resistance to apoptosis, PH is often described as a “tumor-like disease” in recent years (Chan and Rubin, 2017; Marshall et al., 2018). However, despite clear advancement in understanding of the role of apoptosis in hypoxia-associated PH and PH in general, many questions remain unresolved and insufficiently investigated.

A disbalance between pro- and anti-apoptotic molecular pathways has been described as an important phenomenon in the cancer field (Holdenrieder and Stieber, 2004). In addition to the general pathobiology of cellular apoptosis, several circulating apoptotic signals (CASs) have been evaluated as promising biomarkers relevant to the tumor pathologies (Holdenrieder and Stieber, 2010). In the field of cardiovascular diseases, some of the apoptotic markers have been also identified in the blood circulation (Parissis et al., 2004; Niessner et al., 2009). With regard to the pulmonary vascular disease, it has been demonstrated that the levels of CASs, namely Fas ligand (FasL) and tumor necrosis factor (TNF)-related apoptosis-inducing ligand (TRAIL), are changed in response to therapy or altered in patients with PH, respectively (Akagi et al., 2013; Liu et al., 2015). However, almost nothing is known about the profiles of circulating apoptotic markers in the context of human individuals exposed to high altitude hypoxic condition.

Therefore, we investigated for the first time the potential changes in the plasma levels of different apoptotic signals, including FasL, TRAIL and apolipoprotein C1 (ApoC1), in human individuals who live permanently in high altitude regions and lowland subjects who spent a short period of time in such environment. Finally, the potential alteration in gene expression profile of various apoptotic players, for example caspase (Casp) 1 and 3, survivin, Fas-associated death domain protein (FADD)-like ICE inhibitory protein (FLIP), ApoC1, TRAIL and FasL, was analyzed in human PASMCs exposed to hypoxia for different time durations.

## Materials and Methods

### Study Design

In order to analyze the circulating profiles of various apoptotic markers during acute and chronic exposure to high altitude hypoxia, we have performed two studies among the Kyrgyz population.

First, for the purpose of investigating the people permanently living at high altitude environment, we have selected the human community of Sary-Mogol and Achyk-Suu villages, which are located in Alay and Chon-Alay districts of the province of Osh in the southern part of Kyrgyzstan. These villages are settled at altitudes of 3000–3100 m, but the most of the residents usually spend 3–4 months per year at even higher elevations (3200–3600 m). Importantly, we ensured that all subjects enrolled in the study were ethnic Kyrgyz and were born and permanently living at high altitudes. As the lowland control, we have included the volunteers from Bishkek, Kyrgyzstan (approximately 760 m above the sea level). All participants of the study underwent the general anthropometric and echocardiographic measurements (please see below), followed by collection of the peripheral blood and separation of the citrated platelet free plasma (PFP).

**FIGURE 1:**
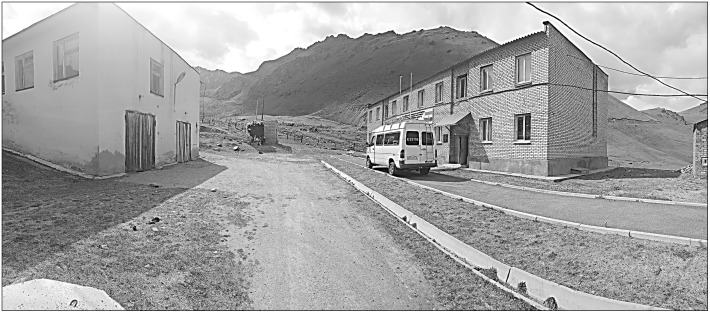
High altitude research station near to the Tuya-Ashuu pass (3200 m), Kyrgyzstan. The photograph is original work of one of the authors.

Secondly, for the purpose of investigating the people acutely exposed to high altitude hypoxia, we had a group of healthy Kyrgyz males. Initially, the subjects underwent examination in Bishkek (low altitude (LA 1) at 760 m, with the outside temperature ranging from 28 to 35°C during the study execution). After that, the participants were transported by road to an altitude of 3200 m (Tuya-Ashuu pass, Kyrgyzstan) (Figure 1), where the outside temperature was in the range 5–20°C, while inside the rooms in the research station the temperature was maintained at 22 ± 2°C. The first 2 days after arrival to high altitude environment the subjects took complete rest. During the next 2 days they were allowed to walk at plains and downhill. Thereafter, the participants were involved in common activities, such as indoor games, watching TV, playing table tennis or billiard, along with morning drill, walking and other galley duties. Importantly, all subjects were free of cardiac or neurological problems and were not consuming any kind of medications. In addition to the basal examination in Bishkek (LA 1) at low altitude, all individuals underwent echocardiographic measurements, followed by the collection of the peripheral blood and separation of the EDTA plasma on days 2 (HA 2), 7 (HA 7), and 20 (HA 20) of high altitude exposure, and on the second day after descent to Bishkek at low altitude again (LA 2). In addition, anthropometry was performed in all participants.

Both studies protocols were approved by the Ethics Committees of the National Centre for Cardiology and Internal Medicine, Bishkek, Kyrgyzstan (01-1/08 and 01-1/07) and the faculty of Medicine at Justus-Liebig University, Giessen, Germany (AZ: 236/16). The study was performed in agreement to the principles outlined in the Declaration of Helsinki of the World Medical Association. Finally, written informed consent was obtained from all participants.

### Anthropometric and Echocardiographic Measurements

Several general parameters were obtained, such as age, gender ratio, body mass index (BMI), and body surface area (BSA). BMI (kg/m^2^) was calculated using the formula: BMI = weight (kg)/(height (m)^2^). BSA (m^2^) was calculated using the Du Bois formula as follows: BSA = 0.007184 × [(height (m) × 100)^0.725^] × (weight (kg)^0.425^). The right ventricular to right atrial pressure gradient was used as a surrogate of the estimated systolic pulmonary artery pressure. Continuous-wave Doppler echocardiography was employed to estimate the tricuspid regurgitant gradient (TRG, in mmHg) from the peak flow tricuspid regurgitation velocity by means of the simplified Bernoulli equation measured using continuous-wave Doppler, as previously described (Yock and Popp, 1984). All the above mentioned parameters are presented in the Table 1 (acute high altitude exposure) and Table 2 (chronic exposure to high altitude). In the case of acute high altitude study, all general parameters, including age, gender, BMI, and BSA were comparable among all participants (Table 1). TRG values were initially increased on the second day (HA 2) of high altitude exposure in comparison to the lowlands (LA 1), and later gradually decreased until return to the low altitude (LA 2) (Table 1). There were statistically significant elevations of TRG on the days 2 and 7 at high altitude, as compared to the lowland conditions (LA 1) (Table 1). Also, there was a significant increase of TRG values on the day 2 of high altitude exposure, in comparison to the low altitude upon return (LA 2) (Table 1).

**Table 1:** Acute exposure to high altitude – human subjects’ anthropometric and echocardiographic data.

				TRG (mmHg)				
				
Age (years)	Gender Ratio m/f(%)	BMI (kg/m^2^)	BSA (m^2^)	LA1	HA 2	HA 7	HA 20	LA 2
25.4 ± 4.2	100/0	23.0 ± 1.9	1.8 ± 0.1	18.5 ± 1.1	23.0 ± 2.2^∗∗∗§§^	20.9 ± 0.8^∗^	19.5 ± 1.6	18.4 ± 1.1


*Adult human subjects who initially came from the lowlands (LA 1) (n = 8) were exposed to high altitude (HA) for 2 (HA 2) (n = 8), 7 (HA 7) (n = 8), and 20 (HA 20) (n = 8) days. After 20 days, they returned to the lowlands again (LA 2) (n = 8). Various anthropometric and echocardiographic parameters at different time points were presented in the table. Results are shown as Mean ± SD (n = 8). ^∗^p < 0.05; ^∗∗∗^p < 0.001 compared to the LA 1. ^§§^ p < 0.01 compared to the LA 2. Friedman test with Dunn’s multiple comparisons test was performed for statistical analysis. m, male, f, female; BMI, body mass index (in kg/m^2^); BSA, body surface area (in m^2^); TRG, tricuspid regurgitant gradient (in mmHg).*

**Table 2:** Chronic exposure to high altitude – human subjects’ anthropometric and echocardiographic data.

	Age (years)	Gender ratio m/f (%)	BMI (kg/m^2^)	BSA (m^2^)	TRG (mmHg)
LA	20.6 ± 2.9	100/0	23.1 ± 1.6	1.9 ± 0.1	20.9 ± 1.6
HA	50.5 ± 11.3	60/40	23.9 ± 4.0	1.7 ± 0.1	20.7 ± 0.4
HA-PH	53.1 ± 14.6	42/58	25.5 ± 5.3	1.7 ± 0.1	46.7 ± 6.0^∗∗∗∗§§§§^


*Adult human subjects permanently living at lowland regions (LA) (n = 10) and high altitude (HA) locations were included for the study. Individuals settled at high altitude regions were separated into two groups: highlanders without pulmonary hypertension (PH) (HA) (n = 10) and highlanders with PH (HA-PH) (n = 12). Various anthropometric and echocardiographic parameters were presented. Results are shown as Mean ± SD (n = 10–12). ^∗∗∗∗^p < 0.0001 LA compared to the HA-PH; ^§§§§^ p < 0.0001 HA compared to the HA-PH. one-way ANOVA with Tukey’s multiple comparisons test was performed for statistical analysis. m, male; f, female; BMI, body mass index (in kg/m^2^); BSA, body surface area (in m^2^); TRG, tricuspid regurgitant gradient (in mmHg).*

In the case of chronic exposure to high altitude (Table 2), BMI and BSA were comparable among all 3 groups: lowland control subjects (LA), highlanders without (HA; TRG ≤ 23 mmHg) and with PH (HA-PH; TRG ≥ 40 mmHg). However, there were noticeable differences in gender ratio and age (lowland control group consisted only of younger males). This fact is a potential limitation of our study. TRG values were significantly increased in highlanders with PH, as compared to the lowland controls and highlanders without PH (Table 2).

### Cell Culture and RT-qPCR

Primary human PASMCs were purchased from Lonza and cultured in Smooth Muscle Growth Medium-2 containing supplement-mix. Human PASMCs passage 7 were incubated at 37°C in a humidified atmosphere of 5% CO_2_ in either normoxic (21% O_2_) or hypoxic conditions (1% O_2_) for a period of 24, 48, and 72 h. Upon the end of the incubation period cells were lysed in RLT buffer (Qiagen) and total cell RNA was extracted using the RNeasy Mini Kit (Qiagen). Complementary DNA (cDNA) was produced by reverse transcriptase polymerase chain reaction via iScript cDNA Synthesis Kit (Bio-Rad). Real-time PCR was performed in Mx3000P qPCR system (Stratagene) using the iTaq Universal SYBR Green Supermix (Bio-Rad). Primer sequences are given from (5^′^ to 3^′^) and presented in the Table 3. Human porphobilinogen deaminase (PBGD) served as a housekeeping gene. In order to confirm specific amplification of the expected PCR product, gel electrophoresis and melting curve analysis were performed.

**Table 3:** Primer sequences are given from (5^′^ to 3^′^).

Target	FP	RP
Human ApoC1	AGC AAG GAT TCA GAG TGC CCC	CCT TCA GCT TAT CCA AGG CAC TG
Human Casp1	TGG GAC TCT CAG CAG ATC AAA CA	GGA TGT GGG CAT AGC TGG GT
Human Casp3	GTA GAA GAG TTT CGT GAG TGC TCG	GCA CAC CCA CCG AAA ACC AG
Human FasL	GGA GAA GCA AAT AGG CCA CCC	CCA GAG GCA TGG ACC TTG AGT
Human FLIP	CGG ACT ATA GAG TGC TGA TGG CA	TCC AAC TCA ACC ACA AGG TCC A
Human survivin	AAA GAG CCA AGA ACA AAA TTG C	GAG AGA GAA GCA GCC ACT GTT AC
Human TRAIL	TCC GTC AGC TCG TTA GAA AGA TGAT	GGT CCC AGT TAT GTG AGC TGC
Human porphobilinogen	CCC ACG CGA ATC ACT CTC AT	TGT CTG GTA ACG GCA ATG CG
deaminase (PBGD)		


*FP, forward primer; RP, reverse primer.*

### Apoptosis and Proliferation Assays

For assessment of apoptosis, human PASMCs (3000 cells per well) were seeded in 96-well white-walled plate in Smooth Muscle Growth Medium-2. After 48 h of recovery time cells were stimulated with different concentrations of SuperFasLigand (Enzo Life Sciences) and incubated at 37°C in water saturated incubators for 24 h under normoxic conditions (21% O_2_). The apoptosis Caspase-Glo 3/7 assay (Promega) was performed following the manufacturer’s instructions. The luminescence of the caspase cleaved substrate reaction was measured after 30 min of incubation at room temperature.

Proliferation of human PASMCs exposed to normoxic (21% O_2_) or hypoxic conditions (1% O_2_) was assessed by Cell Proliferation ELISA, 5-bromo-2^′^-deoxyuridine (BrdU) assay (Sigma Aldrich). In more details, 7500 cells per well were seeded into 24-well plates and following the 24 h starvation period PASMCs were incubated for 24 or 48 h in Smooth Muscle Growth Medium-2. After that the cells were treated with different concentrations of SuperFasLigand or 500 ng/ml of neutralizing anti-Fas antibody ZB4 (Millipore). In addition, PASMCs were incubated for 48 h in Smooth Muscle Basal Medium and stimulated with 50 ng/ml of platelet-derived growth factor (PDGF) (R&D Systems) and/or 500 ng/ml of neutralizing anti-Fas antibody ZB4. The absorbance of the substrate reaction was measured at 370 nm (reference wavelength 492 nm).

### Enzyme-Linked Immunosorbent Assay (ELISA)

Citrated platelet free plasma (PFP) or EDTA plasma samples were obtained from the peripheral blood from all participants of the study. In order to analyze the circulating levels of different markers, several ELISA measurements were performed for the following targets (ELISA kits): ApoC1 (Abnova), TRAIL (R&D Systems), FasL (LSBio/R&D Systems) and B-type natriuretic peptide (BNP) (Abnova). The concentrations of the markers were expressed as μg or pg per mL of plasma.

### Data Analysis

Results are presented as Mean ± SD. Unpaired *t*-test with Welch’s correction or ordinary one-way ANOVA with Dunnett’s or Tukey’s multiple comparisons test were used to compare data derived from the cell culture. Data based on ELISA and echocardiographic measurements were analyzed using Friedman test with Dunn’s multiple comparisons test, RM one-way ANOVA with Tukey’s multiple comparisons test or ordinary one-way ANOVA with Tukey’s multiple comparisons test. Spearman or Pearson tests were used for the correlation analysis. Statistical significance was considered when *p*-value was < 0.05, < 0.01, < 0.001, and < 0.0001.

## Results

### Alteration in the Gene Expression Levels of Various Apoptotic Markers in Human PASMCs After Hypoxic Incubation

**FIGURE 2:**
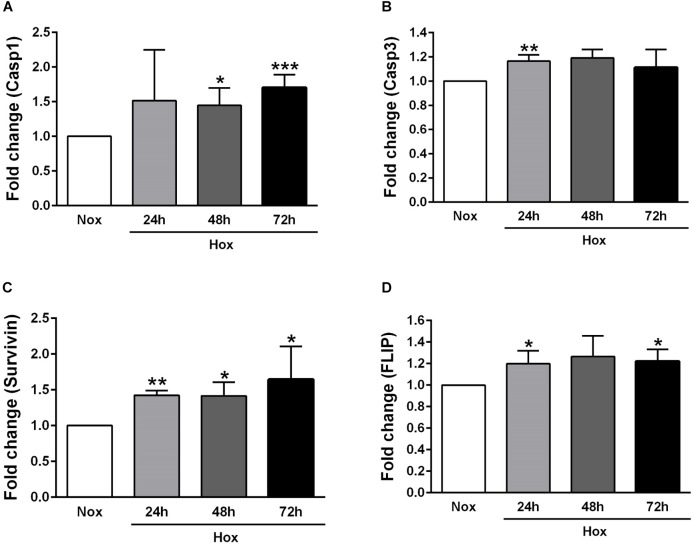
Gene expression profiles of different apoptotic markers in human pulmonary arterial smooth muscle cells (PASMCs) exposed to hypoxia I. Human PASMCs were exposed to hypoxia (Hox) for different time durations (24, 48, and 72 h), followed by RT-qPCR for measurement of various apoptotic markers, such as: **(A)** caspase 1 (Casp1) and **(B)** 3 (Casp3), **(C)** survivin, and **(D)** Fas-associated death domain protein (FADD)-like ICE inhibitory protein (FLIP). Normoxia (Nox) exposure served as a control. Results are presented as a fold change normalized to the respective normoxic controls. Results are expressed as Mean ± SD (*n* = 4). ^∗^*p* < 0.05, ^∗∗^*p* < 0.01; ^∗∗∗^*p* < 0.001 Nox versus Hox. Unpaired *t*-test with Welch’s correction was performed for statistical analyses.

**FIGURE 3:**
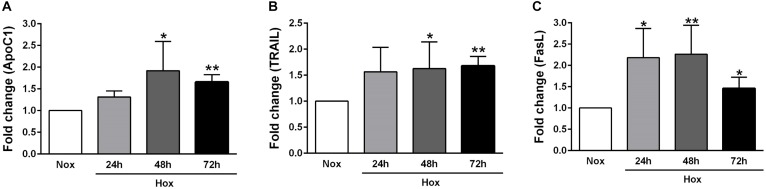
Gene expression profiles of different apoptotic markers in human PASMCs exposed to hypoxia II. Human PASMCs were exposed to hypoxia (Hox) for different time durations (24, 48, and 72 h), followed by RT-qPCR for measurement of various apoptotic markers, such as: **(A)** apolipoprotein C1 (ApoC1), **(B)** tumor necrosis factor (TNF)-related apoptosis-inducing ligand (TRAIL), and **(C)** Fas ligand (FasL). Normoxia (Nox) exposure served as a control. Results are presented as a fold change normalized to the respective normoxic controls. Results are expressed as Mean ± SD (*n* = 4). ^∗^*p* < 0.05; ^∗∗^*p* < 0.01 Nox versus Hox. Unpaired *t*-test with Welch’s correction was performed for statistical analyses.

As already described in Section “Materials and Methods,” human PASMCs were exposed to hypoxia condition for different time durations (24, 48, and 72 h), followed by quantification of the gene expression levels of various apoptotic markers, such as Casp1 and 3, survivin, FLIP, ApoC1, TRAIL, and FasL (Figure 2, 3). In general, all investigated apoptotic signals had increased their expression profiles upon hypoxic stimuli in human PASMCs (Figure 2, 3), as compared to the normoxia condition. However, there were no clear and uniform time-dependent changes in the expression profiles of investigated apoptotic markers. The most prominent and statistically significant upregulation was noticed in the case of survivin and FasL, during all time points of hypoxia exposure, in comparison to the respective normoxic controls (Figure 2C, 3C).

### Circulating Profiles of Apoptotic Markers in Human Subjects Exposed to Acute High Altitude Conditions

**FIGURE 4:**
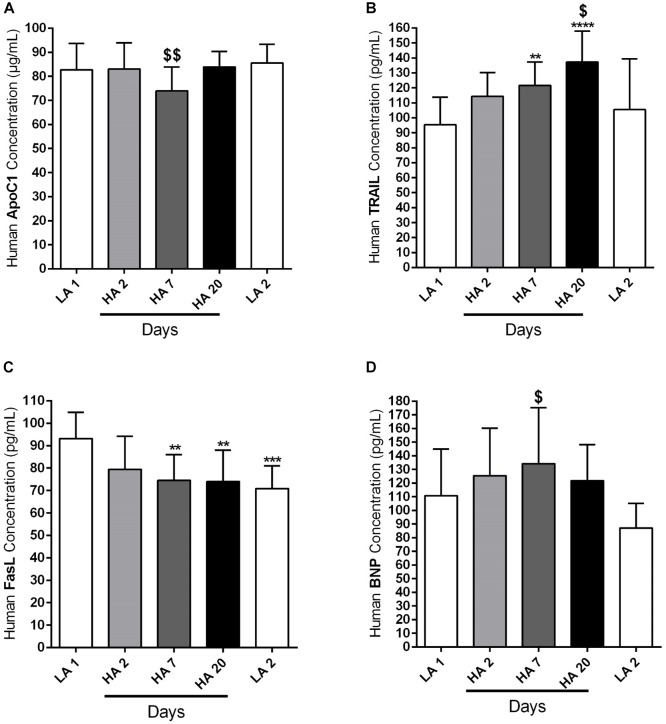
Circulating apoptotic markers in human subjects during acute exposure to high altitude. Healthy volunteers living at lowland regions of Kyrgyzstan (LA 1) (*n* = 7–8) were exposed to high altitude (HA) environment (3200 m) in total duration of 20 days. After exposure to this extreme environment they returned to the lowlands again (LA 2) (*n* = 8). Echocardiographic measurements and collection of the peripheral blood were performed during the following time points: in low altitude location (LA 1), after 2 (HA 2) (*n* = 8), 7 (HA 7) (*n* = 8), and 20 (HA 20) (*n* = 8) days spending at high altitude, and after return to the lowlands again (LA 2). Plasma was separated and enzyme-linked immunosorbent assay (ELISA) was performed for the detection and quantification of the following circulating apoptotic markers: **(A)** apolipoprotein C1 (ApoC1), **(B)** TNF-related apoptosis-inducing ligand (TRAIL), and **(C)** Fas ligand (FasL). In addition, the circulating profile of B-type natriuretic peptide (BNP) was analyzed by ELISA. **(D)** Results are expressed as concentrations of the above mentioned markers (in μg or pg per mL of plasma) and presented as Mean ± SD (*n* = 7–8). ^∗∗^*p* < 0.01; ^∗∗∗^*p* < 0.001; ^∗∗∗∗^*p* < 0.0001 compared to the LA 1 group. ^$^*p* < 0.05; ^$$^*p* < 0.01 compared to the LA 2 group. Friedman test with Dunn’s multiple comparisons test, RM one-way ANOVA with Tukey’s multiple comparisons test or ordinary one-way ANOVA with Tukey’s multiple comparisons test were performed for statistical analyses.

We have already mentioned in the Section “Materials and Methods” that circulating levels of different apoptotic markers, such as ApoC1, TRAIL and FasL, were measured by ELISA in the plasma samples of human volunteers taken at different time points: in the lowland conditions (LA 1), on days 2 (HA 2), 7 (HA 7), and 20 (HA 20) of high altitude exposure, and after return to the low altitude (LA 2). In addition, ELISA was performed in the plasma samples during the same time points in order to analyze the level of circulating BNP. Investigated circulating apoptotic markers had different expression profiles and due to the technical reasons not all values for all enrolled subjects are available. Apo C1 circulating levels (in μg/mL) were comparable among different time points, except on day 7 (HA 7) of high altitude exposure, when the levels of this marker were significantly decreased in comparison to the low altitude upon descent from the high altitude (Figure 4A). Further, circulating levels of TRAIL (in pg/mL) were gradually increasing due to high altitude exposure, with being significantly enhanced on days 7 (HA 7) and 20 (HA 20), in comparison to the lowland conditions (LA 1) (Figure 4B). After returning to the lowlands again, there was a significant decrease of circulating TRAIL levels (Figure 4B). In the case of FasL (in pg/mL), there was clear, but not time-dependent reduction of the circulating levels of this marker in high altitude conditions, as compared to the lowlands (LA 1) (Figure 4C). Surprisingly, decreased levels of circulating FasL were kept upon return to the low altitude (Figure 4C). Finally, there was a noticeable trend of increased levels of circulating BNP (in pg/mL) during different time points at high altitude, with prominent reduction upon return to the lowlands (Figure 4D). However, the significant difference was present only between the circulating levels of this marker on day 7 (HA 7) and the second low altitude condition (LA 2) (Figure 4D).

### Circulating Profiles of Apoptotic Markers in Kyrgyz Highlanders and Lowlanders

**FIGURE 5:**
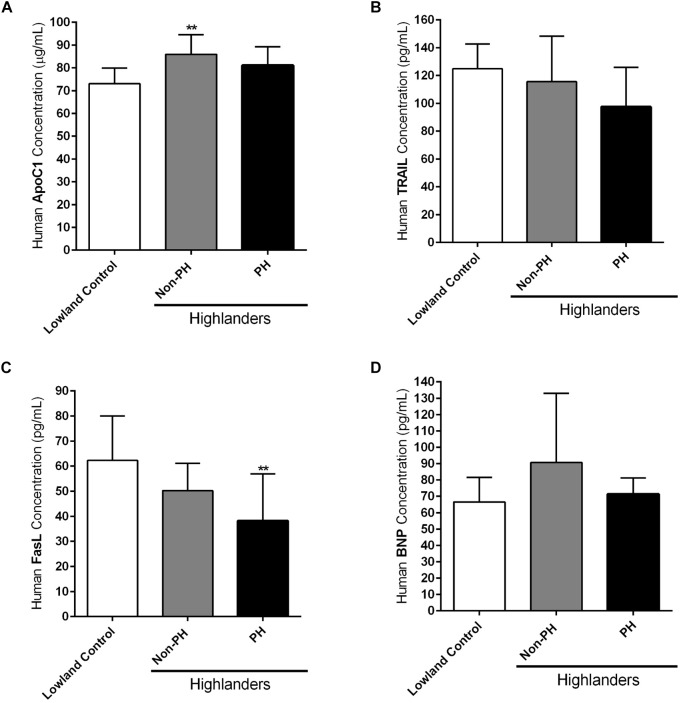
Circulating apoptotic markers in human subjects permanently living at high altitude. Human subjects permanently living at high altitude regions of Kyrgyzstan (highlanders) were separated into two groups: individuals without developed pulmonary hypertension (Non-PH) (*n* = 9–10) and individuals with this pulmonary vascular disease (PH) (*n* = 10). People living at the low altitude served as a control (*n* = 9–10). Echocardiographic measurements and collection of the peripheral blood were performed for all volunteers. Plasma was separated and enzyme-linked immunosorbent assay (ELISA) was performed for the detection and quantification of the following circulating apoptotic markers: **(A)** apolipoprotein C1 (ApoC1), **(B)** TNF-related apoptosis-inducing ligand (TRAIL), and **(C)** Fas ligand (FasL). In addition, the circulating profile of B-type natriuretic peptide (BNP) was analyzed by ELISA. **(D)** Results are expressed as concentrations of the above mentioned markers (in μg or pg per mL of plasma) and presented as Mean ± SD (*n* = 9–10). ^∗∗^*p* < 0.01 compared to the lowland control. One-way ANOVA with Tukey’s multiple comparisons test was performed for statistical analysis.

As already indicated in the Section “Materials and Methods,” circulating levels of different apoptotic markers, such as ApoC1, TRAIL and FasL, were measured by ELISA in the plasma samples of human subjects permanently living at high altitudes, in comparison to the people settled in the lowland locations (Lowland Control). Highlanders were further divided into two groups, those who developed PH (PH) and those who did not develop this pulmonary vascular disease (Non-PH). In addition, ELISA was performed in the plasma samples of these three groups, in order to analyze the level of circulating BNP. Due to the technical reasons not all values for all enrolled subjects are available. ApoC1 circulating levels (in μg/mL) were increased in both highlander groups, with being statistically significant in the case of highlanders without PH, in comparison to the lowland controls (Figure 5A). TRAIL circulating profile (in pg/mL) did not reveal significant changes among groups, however, there was a trend of reduction in the level of this marker in highlanders with PH, as compared to the people living at low altitude (Figure 5B). Further, there was a visible decrease in the circulating levels of FasL (in pg/mL) in both highlander groups, with statistically significant alteration in highlanders with PH, in comparison to the lowland control (Figure 5C). Finally, there were no significant changes in the context of BNP (in pg/mL) among different groups (Figure 5D). Surprisingly, there was a trend of elevated levels of circulating BNP in highlanders without PH, as compared to other two groups (Figure 5D).

### Circulating FasL Levels Negatively Correlate With TRG Values in Kyrgyz Population

**FIGURE 6:**
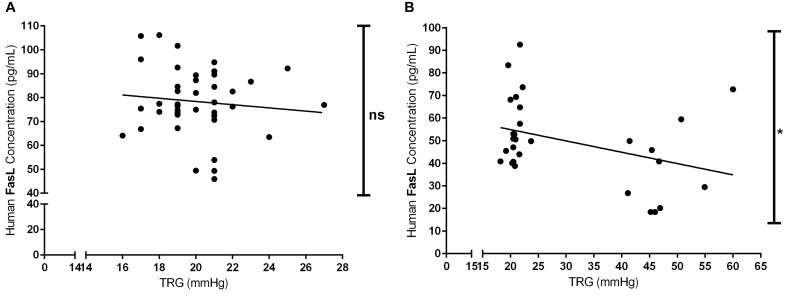
Correlations between the circulating levels of Fas ligand and tricuspid regurgitant gradient (TRG) during acute and chronic exposure to high altitude. Correlations between the concentrations of circulating Fas ligand (FasL, in pg/mL) and TRG (in mmHg) values in human subjects exposed to acute **(A)** and chronic **(B)** effects of high altitude environment are shown. Spearman or Pearson tests were performed for statistical analyses. ^∗^*p* < 0.05; ns, not significant.

Furthermore, we have investigated the correlation between the circulating levels of FasL (pg/mL) in both acute and chronic exposure of Kyrgyz volunteers with TRG values (Figure 6). Although there was a slight trend, there was no significant correlation between these two parameters in human subjects exposed acutely to high altitude environment (Figure 6A). However, there was a significant negative correlation between circulating levels of FasL and TRG in permanent residents of lowlands and highlands in investigated Kyrgyz population (Figure 6B).

### FasL Exerts Pro-apoptotic and Anti-proliferative Effects in Human PASMCs

**FIGURE 7:**
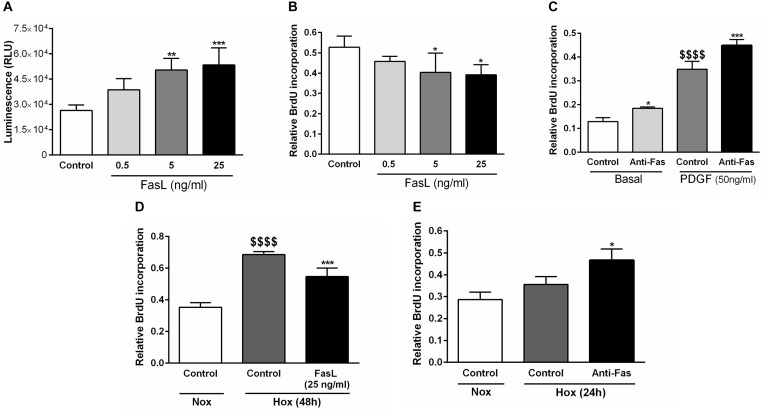
Pro-apoptotic and anti-proliferative functions of Fas ligand in human PASMCs. Human PASMCs were exposed to normoxic (Nox) **(A–C)** or hypoxic (Hox) **(D,E)** conditions and treated with different concentrations (ng/ml) of Fas ligand (FasL) or anti-Fas, followed by measurements of cellular apoptosis and proliferation, using apoptosis assay **(A)** and 5-bromo-2^′^-deoxyuridine (BrdU) incorporation assays **(B–E)**, respectively. Results are presented as Mean ± SD (*n* = 4). ^∗^*p* < 0.05; ^∗∗^*p* < 0.01; ^∗∗∗^*p* < 0.001 compared to the respective controls. ^$$$$^*p* < 0.0001 basal/normoxic control compared to the PDGF/hypoxic control, respectively. Ordinary one-way ANOVA with Dunnett’s or Tukey’s multiple comparisons test was performed for statistical analysis. PDGF, platelet-derived growth factor.

Finally, we have analyzed the relevant cellular functions of FasL in human PASMCs, such as apoptosis and proliferation (Figure 7). We have found that different concentrations (5 and 25 ng/ml) of FasL significantly increased apoptosis of PASMCs (Figure 7A). Furthermore, the same concentrations of this ligand significantly reduced the proliferation of PASMCs under normoxic conditions (Figure 7B). In addition, anti-Fas enhanced the cellular proliferation under the basal conditions as well as during the stimulation with PDGF (Figure 7C). PASMCs were also exposed to hypoxic conditions (Figure 7D,E) and FasL (25 ng/ml) significantly decreased the proliferation of these cells (Figure 7D). Finally, anti-Fas resulted in augmentation of the PASMCs proliferation (Figure 7E). Clearly, FasL demonstrates pro-apoptotic and anti-proliferative properties in human PASMCs.

## Discussion

In general, the findings of our study revealed that:

(1) There was a prominent enhancement in gene expression of various apoptotic players in human PASMCs exposed to hypoxia condition,
(2) There were differential changes in investigated circulating apoptotic markers during acute and chronic exposure to high altitude in humans, with the most uniform alteration in FasL,
(3) FasL levels in plasma negatively correlated with TRG values in Kyrgyz population permanently settled in low and high altitudes, and,
(4) FasL exerted pro-apoptotic and anti-proliferative functions in human PASMCs.

Cellular apoptosis is an important, complicate and complex process (Sartorius et al., 2001). Under various stimuli, caspases’ may activate and perform the apoptosis (Sartorius et al., 2001). Results of our study demonstrated an augmentation in gene expression of Casp 1 and 3 after the hypoxia incubation in PASMCs. Despite the fact that PH in general is characterized by “apoptosis-resistant” events, the increase in Casp3 expression was found in the lungs of monocrotaline model and the interpretation has to be taken with caution, since there are also some evidences that caspases may even exert anti-apoptotic features (Sartorius et al., 2001; Yang and Widmann, 2001; Le Goff et al., 2012; Hong et al., 2014).

With regard to other apoptotic players, our study indicated an increase in gene expression profiles of ApoC1, FLIP, and survivin in PASMCs exposed to hypoxia, in comparison to the respective normoxic controls. The literature suggested that ApoC1, FLIP, and survivin are generally considered as anti-apoptotic signals in different conditions (Sartorius et al., 2001; Takano et al., 2008; Portt et al., 2011; Fan et al., 2015; Zhang et al., 2015, 2016). Importantly, survivin is well-investigated in the context of hypoxia-induced PH and our data are in line with the literature sources (Fan et al., 2015; Zhang et al., 2015, 2016).

In the case of TRAIL and FasL, we have shown a noticeable increase in the gene expression for both targets during the hypoxic exposure of PASMCs, as compared to their respective normoxic controls. Limited evidences from the literature implicated these two signals in the context of pulmonary vascular disease (Hameed et al., 2012; Saito et al., 2015). Interestingly, although TRAIL is usually considered as pro-apoptotic mediator, its role in experimental PH pathogenesis has been described (Hameed et al., 2012). Taken together, it is clear that there is a complex play of different apoptotic mediators and this cellular process is indeed dysregulated in the pulmonary vascular cells exposed to hypoxia, however, it goes beyond the simplistic explanation based only on the expression patterns (increased/decreased) or historically known pro- or anti-apoptotic features of these signals.

In the cancer field, abnormally regulated apoptosis causes the release of apoptotic products into circulation, and they may represent potential biomarkers (Holdenrieder and Stieber, 2010). Up to date, there is no evidence about the expression profile of circulating apoptotic markers in humans exposed to acute and chronic effects of high altitude. In our study, we have investigated the concentration of ApoC1, TRAIL, and FasL in the plasma of Kyrgyz lowlanders who spent a short period of time in high altitude environment. The same signals were explored in the circulation of the permanent dwellers of high altitude who did or did not develop the HAPH, as well as in the blood of lowlanders. In addition, we have analyzed the circulating BNP, which is usually considered as an important cardiac biomarker, relevant to the PH field (Mellor et al., 2014; Anwar et al., 2016). Briefly, BNP has shown a trend to initially increase with days spent at high altitude in the circulation of Kyrgyz volunteers, as compared to the lowland conditions, and after day 7 there was a tendency to decrease, with a visible reduction upon descent to the lowlands again. Similarly, there was no clear change in the circulating profile of this cardiac marker in highlanders compared to the lowlanders, except non-significant augmentation in highlanders without PH.

ApoC1 circulatory levels were mostly comparable during the acute exposure of subjects to high altitude conditions, while there was some signal of increased values of this marker in the circulation of highlanders, in comparison to lowlanders. Yet, the data do not convincingly suggest the significant alteration of ApoC1 neither during acute nor chronic exposure to high altitude conditions.

Interestingly, the circulating concentrations of TRAIL showed the opposite manner in acute versus chronic exposure to high altitude in human volunteers. On one hand, there was a clear augmentation of this marker with days spent at high altitude in the blood of lowlanders, with significant reduction upon descent to low altitude again. On the other hand, there was noticeable, yet insignificant reduction of the circulating levels of TRAIL in highlanders (particularly those who developed HAPH) in comparison to the lowlanders. Furthermore, the literature suggested the elevated values of this marker in PH patients (Liu et al., 2015). Therefore, further studies are crucially needed to reveal the precise role of this apoptotic signal in both acute and chronic exposure to high altitude.

Finally, clearer and more informative results were obtained with focus on FasL in the circulation of humans in both acute and chronic exposure to high altitude geographic locations. There was a significant reduction of circulating FasL values in lowlanders exposed to high altitude environment for a short period of time. In addition, the circulating concentrations of this apoptotic marker were significantly decreased in highlanders with PH, as compared to the lowlanders. Interestingly, there were attenuated values of FasL in highlanders without PH also, in comparison to the lowland control, however, the change was not statistically significant. Lastly, there was a negative correlation between the circulatory levels of FasL and TRG, clearly indicated the potential biomarker properties of this apoptotic signal in the case of chronic effects of high altitude and HAPH. In accordance with our findings, FasL serum values were shown to be relevant to the field of pulmonary vascular diseases, as evident in the literature (Akagi et al., 2013; Saito et al., 2015). Importantly, FasL expression was increased in PASMCs derived from patients with idiopathic pulmonary arterial hypertension upon the treatment with prostaglandin I2, which was associated with induction of apoptosis (Akagi et al., 2013). Following this line of thinking, we have also demonstrated in this study that FasL enhanced the apoptotic process in PASMCs. Consistently, pro-apoptotic features of FasL in PASMCs were described in the literature (Zhang et al., 2003; Wang et al., 2010). Furthermore, we have shown that the same concentrations of FasL used for the assessment of apoptosis significantly reduced the proliferation of these pulmonary vascular cells under normoxic conditions. In addition, the blockage of FasL resulted in increased proliferation of PASMCs under the basal conditions as well as during the stimulation with PDGF. Of note, PDGF signaling is a very well-known player associated with hypoxia and pulmonary vascular remodeling (Schermuly et al., 2005; ten Freyhaus et al., 2011; Veith et al., 2014). Finally, we have demonstrated that FasL exerted anti-proliferative effects on PASMCs exposed to hypoxic conditions, and blockage of FasL resulted in enhancement of the proliferation process.

Overall, our study identified for the first time that circulating levels of apoptotic signal FasL are reduced during acute and chronic exposure to high altitude environment. In addition, due to its pro-apoptotic and anti-proliferative functions in the relevant cells of the pulmonary vasculature, dysregulated FasL signal may play an important role in the context of HAPH.

## Author Contributions

DK, HG, ASa, and RS conceived, designed, and created the study. DK, SP, AP, ASy, AbM, ArM, MS, KMU, MC, AT, NO, MD, and CV organized the expeditions and performed the field and laboratory measurements. DK, ASa, AbM, HG, ASy, and RS drafted and wrote the manuscript. OP and NW contributed with significant intellectual content. All authors read and approved the manuscript.

## Conflict of Interest Statement

The authors declare that the research was conducted in the absence of any commercial or financial relationships that could be construed as a potential conflict of interest.
